# Integral definition and conceptual model of mental health: Proposal from a systematic review of different paradigms

**DOI:** 10.3389/fsoc.2022.978804

**Published:** 2022-11-25

**Authors:** Maday Alicia Coronel-Santos, Juan Carlos Rodríguez-Macías

**Affiliations:** ^1^School of Humanities and Education, Tecnológico de Monterrey, Monterrey, Mexico; ^2^Institute of Research and Educational Development, University of Baja California, Ensenada, Mexico

**Keywords:** mental health, wellbeing, self-care, mental disorders, state of balance, systematic literature review (SLR), explanatory model

## Abstract

Global society presents a mental health scenario characterized by the prevalence of mental disorders and the limited existence of formal care services. Faced with such a context, it is necessary to review what is understood and done in favor of mental health. This implies, in the first instance, analyzing the concept of mental health from a comprehensive approach that takes into account different perspectives from the social and natural sciences, related factors, and care options. Therefore, the present work aimed to propose an integral definition and a conceptual model of mental health based on the Systematic Literature Review, with the PRISMA approach, of the theoretical frameworks of 52 articles related to mental health published up to February 2022. A qualitative approach was used, with a Grounded Theory design, which allowed comparing different paradigms and identifying gaps in conceptual notions to build an explanatory model of mental health. The results showed three dominant paradigms that circumscribe the concept of mental health. Mental health is understood as the absence of illness, positive mental health, and a state of equilibrium. In addition, the need to propose a definition that integrates these dominant paradigms was mainly identified, and that would allow a broader understanding of the state of equilibrium as the basic process through which the individual must pass in the search for personal recovery. From the comparative analysis of the categories designated according to the Grounded Theory approach, an explanatory model was proposed to define mental health as the internal process of self-care, centered on the self-awareness and self-regulation of the human being, in which the person seeks to balance their feelings, thoughts, and behaviors, intrapersonal and interpersonal ones, to approach an optimal state of wellbeing and absence of mental disorders according to universal values and symptoms, and biological, social, psychological, and environmental factors.

## Introduction

The global society presents a mental health landscape characterized by the prevalence of mental disorders and the limited existence of formal care services; given that 5% of adults suffer from depression, one in five children and adolescents has a mental disorder, and only 13 out of every 100,000 inhabitants are mental health care professionals [(World Health Organization (WHO), 2019, 2021)]. Given such a scenario, it is necessary to reflect on what is understood and done in favor of mental health, which implies, in the first instance, analyzing the concept from an integral approach that contemplates diverse perspectives of natural and social sciences, related factors, and care options. The result of this analysis provides a reformulation of strategies for mental health promotion, prevention, and care.

Regarding several perspectives, Busfield (2000) offers a concomitant view between the natural and social sciences, which seeks to understand mental health, providing evidence of the importance of both perspectives. On one hand, she argues that the natural sciences tend to analyze the nature of the body and its bodily processes, and explain mental health from mental disorders and their diagnostic symptoms [e.g., the Diagnostic and Statistical Manual of Mental Disorders (American Psychiatric Association, 2013)]. In contrast, social sciences lean toward the analysis of thoughts and behaviors using norms and values that are socially designated. In this sense, Durkheim (1964), through his sociological study, showed that the guidelines that define the pathological from the natural sciences contribute to reinforcing the values of society; that is, sociological studies have paid attention to the concepts of mental disorder and diagnostic symptoms to define what is acceptable behavior and action within society. In this sense, it is necessary to show how interdisciplinary and multidisciplinary efforts between natural sciences (biology, neuroscience, medicine, etc.) and social sciences (psychology, sociology, philosophy, economics, etc.) converge to give meaning to mental health.

With respect to the factors related to mental health, the following aspects can be considered: biological (age, sex, genetics, and special conditions), psychological (personality traits, values, motivations, and aspects of self-regulation), social (educational level, gender, socioeconomic status, marital status, occupation, and family composition), and environmental (stressful, challenging, hostile, among others); by the contributions of several authors [for example, DeNeve and Cooper, 1998; Lyubomirsky et al., 2005; Keyes et al., 2010; American Psychiatric Association, 2013; Lim, 2017; World Health Organization (WHO), 2018; among others].

In relation to mental health care options, World Health Organization (WHO) (2009) made a distinction between informal services (self-care and community care), and formal services (primary care, community services, psychiatric in general hospitals, long-stay facilities, and specialized services). However, it emphasized that self-care is essential and occurs simultaneously with other services, as the person self-manages, without the intervention of a professional, their mental health problems; in addition to promoting and fostering their recovery, and better mental health.

Based on the above, it is clear that mental health is a complex concept, which, from different approaches, has given rise to a proliferation of conceptual notions, making it difficult to measure and create strategies for improvement. In this sense, it is necessary to reduce the gap by analyzing the input toward a proposal for a unifying concept. Therefore, the aim of the present study was to analyze different conceptual contributions regarding mental health through the Systematic Literature Review (SLR).

Literature reviews related to the definition of mental health are few, mostly focused on analyzing specific related aspects, for example, mental health in specific populations (Suhaiban et al., 2019), the type of care people receive (Bakker et al., 2016), related factors (Shalaby and Agyapong, 2020), among others. The concept of mental health was identified by the work of Muñoz et al. (2016), which was based on an analysis of positive mental health, and the work done by Wang and Lai (2022), in which they used two paradigms as references in their analysis: the absence of disease and positive mental health. As can be seen, the literature reviews on mental health are diverse and polysemic. In this scenario, the present study is based on the SLR that identifies, analyzes, and evaluates the theoretical references of various disciplines, from a holistic, interdisciplinary, and multidisciplinary approach to synthesize the conceptual approaches of different perspectives toward the construction of an integral concept of mental health.

In summary, this study aimed to propose an integral definition and conceptual model of mental health based on the SLR of the theoretical frameworks used in articles related to mental health published up to February 2022. We analyzed (1) the accepted theoretical references on mental health; (2) the dominant conceptual approaches (paradigms) in the field of mental health; and (3) the gaps identified in the conceptual notions of mental health.

## Materials and methods

The research was developed through a qualitative approach, with a Grounded Theory design, based on SLR. It focused on the Grounded Theory (Glaser and Strauss, 1967) because, through the comparative analysis of theoretical frameworks, different paradigms were identified and analyzed to provide a comprehensive model of mental health. The comparative analysis was guided by a systematic method (SLR), which allows the identification, interpretation, and evaluation of scientific productions in an area of interest during a specific period (Fink, 1998).

The process developed was inspired by the SLR conducted by Kitchenham and Charters (2007) and the PRISMA approach (Moher et al., 2009). The application of this methodology enabled the search for information to be narrowed down following the objectives set. The steps used were: (1) definition of the objective and research questions, (2) search strategy, (3) inclusion and exclusion criteria, (4) selection of studies, and (5) data analysis strategy.

### Definition of the objective and research questions

The first step consisted of defining the purpose of the SLR to direct the review method. The main objective was to propose an integral definition and a conceptual model of mental health, based on the analysis of the theoretical references used in articles related to mental health. To fulfill the stated objective, a set of questions were defined to guide the investigation:

Q1: what are the accepted theoretical references regarding mental health from its historical approach?
Q2: what are the dominant conceptual approaches (paradigms) in the field of mental health?
Q3: what are the gaps identified in the conceptual notions of mental health?

### Search strategy

At this stage, the databases to be reviewed and the search string were defined. The databases used were Scopus and Web of Science (WoS), as they are considered among the main multidisciplinary bibliographic databases. According to the research questions and the contributions of Muñoz et al. (2016) and Wang and Lai (2022), keywords were identified and incorporated into the search string of each database. To include relevant studies, Boolean operators were used. Each database was searched for articles related to the following keywords: “Mental health,” “Mental well^*^,” “Social well^*^,” “Emotional well^*^,” and “Psychological well^*^”. The main objective was to analyze scientific products that examined these elements as a whole and not in isolation, so it was decided to use the Boolean operator AND instead of OR, which considerably delimited the initial study sample. Subsequently, filters were applied concerning the type of document (article, review, and book chapter) and language (English and Spanish); the area of study was not filtered since the objective implied analyzing the contributions of various disciplines.

### Inclusion and exclusion criteria

From the search results in each database, inclusion and exclusion criteria were applied to refine the results. Articles that included the term mental health or wellbeing in the title or abstracts were considered. Articles that could not be accessed in the full text were excluded, and subsequently, the theoretical frameworks of the resulting products were analyzed and those that did not include conceptual notions about mental health were discarded.

### Selection of studies

**Figure 1:**
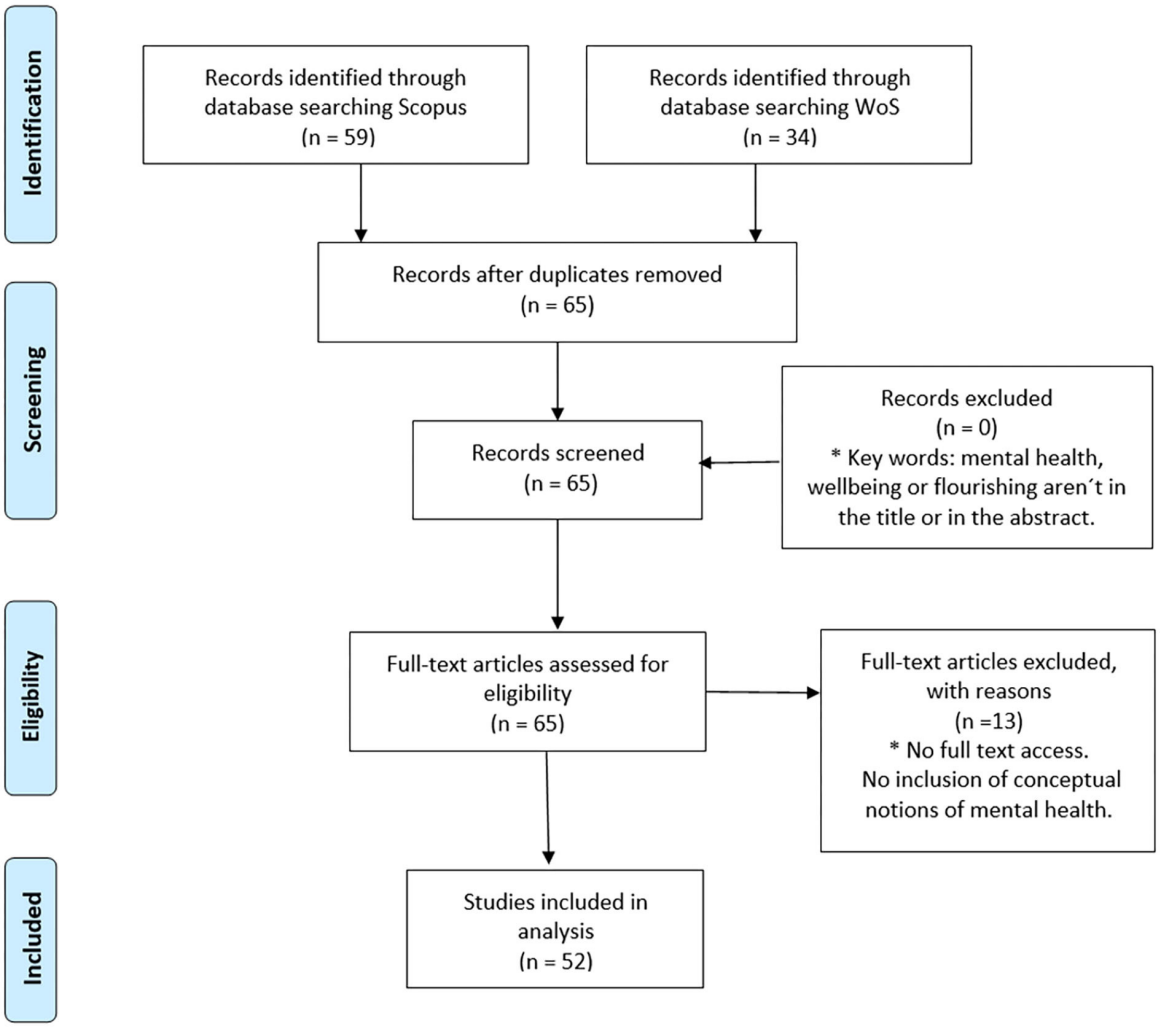
The article selection process for SLR [Adapted from Moher et al. (2009)].

According to the PRISMA approach (Moher et al., 2009), a flow chart was prepared (Figure 1), which shows the results of the selection process of articles that were reviewed. The *Identification* phase shows the total number of articles obtained per database consulted, according to the keywords and filters applied. During the *Screening* phase, the databases were combined, duplicates were eliminated, and inclusion criteria were applied according to the titles and abstracts of the articles. In the *Eligibility* phase, the theoretical frameworks of the scientific products were analyzed, and exclusion criteria were applied. Finally, during the *Included* phase, the articles that were analyzed were determined. Figure 1 shows the flow chart applied for the present SLR, which resulted in a total of 52 scientific articles.

### Data analysis strategy

Based on the selected articles, we proceeded to read each article with special attention to the theoretical framework to identify theoretical–conceptual references on mental health. Subsequently, with the information collected, a comparative analysis was carried out using the designation of categories from the Grounded Theory approach (Strauss and Corbin, 1990): open coding (assignment of codes or labels), axial coding (classification of codes into categories), and selective coding (identification of central category), which served as input for the design of the explanatory model.

## Results

### Q1: What are the accepted theoretical references regarding mental health from its historical approach?

The theoretical frameworks used in the articles reviewed were analyzed and the accepted theoretical references regarding mental health were identified and are shown in Table 1. The definition of mental health has changed over time. Initially, from philosophical currents, the concept was approached from the study of happiness, years later, from medicine, it was considered as the absence of disease, which was consolidated with the Diagnostic and Statistical Manual of Mental Diseases (American Psychiatric Association, 1952). Later, from the perspective of positive psychology, philosophical contributions were reconsidered, and mental health was defined as wellbeing and happiness, an approach that has been built over the years with the contributions of various research studies. In recent years, a new approach has emerged from medicine and psychiatry as a critique of mental health based on wellbeing and happiness, which conceptualizes mental health as a dynamic state of internal balance.

**Table 1:** Accepted theoretical referents regarding mental health.

**Year**	**Authors**	**Contribution**
4th century B.C.	Aristippus (Alonso, 2017)	Hedonism.
4th century B.C.	Aristotle (Castelló, 1993)	Eudemia.
1729	Nicholas Robinson (Salaverry-García, 2012)	Mental illness treated by medicine.
1793	Philippe Pinel (Woods and Carlson, 1961)	Scientific Psychiatry.
1941	Sigerist (1941)	Health beyond illness.
1952	American Psychiatric Association (1952)	Diagnostic and Statistical Manual of Mental Disorders (DSM-I).
1958	Jahoda (1959)	Positive mental health: attitudes of an individual toward his or herself; growth, development, and self-realization; personality integration; autonomy, perception of reality; and mastery of the environment.
1969	Bradburn (1969)	Psychological wellbeing: preponderance of positive feelings.
1984	Diener (1984)	Subjective wellbeing: life satisfaction and preponderance of positive feelings.
1988	Watson et al. (1988)	Affection: positive affect and negative affect.
1989	Ryff (1989)	Psychological wellbeing: self-acceptance, purpose in life, autonomy, positive relationships, mastery of the environment and personal growth.
1993	Waterman (1993)	Happiness: experiences of personal expressiveness (self-realization) and of hedonic enjoyment (pleasure).
1998	Keyes (1998)	Social wellbeing: social acceptance, social integration, social contribution, social coherence and social actualization.
2001	Greenspoon and Saklofske (2001)	Dual factor model of mental health: absence of mental illness and positive mental health based on wellbeing.
2002	Keyes (2002)	Emotional wellbeing (positive affect and absence of negative affect), psychological wellbeing
		(Ryff, 1989) and social wellbeing (Keyes, 1998).
2005	Keyes (2005)	Two continuum models of mental illness and mental health: disease and flourishing (emotional, psychological and social wellbeing).
2009	Huppert and So (2009)	Mental health spectrum: mental illness (depression and anxiety) and flourishing (positive emotions, self-esteem, optimism, vitality, self-determination and positive relationships).
2011	Seligman (2011)	Flourishing: positive emotion, engagement, relationships, sense and achievement.
2012	Diener and Tov (2012)	Emotions and mood (positive and negative emotions), cognitive judgments, and motivational aspects (engagement, optimism, confidence and positive energy).
2012	Dambrun et al. (2012)	Happiness: self-centered psychological (positive feelings of high exaltation) Selfless psychological functioning (feelings of peace of mind).
2015	Galderisi et al. (2015)	Dynamic state of internal equilibrium: basic cognitive skills, basic social skills, emotion regulation, empathy, flexibility, resilience in the face of distress, and harmonious relationship between body and mind.

Own elaboration.

### Q2: What are the dominant conceptual approaches (paradigms) in the field of mental health?

From the analysis of the accepted theoretical references, three dominant paradigms were identified that circumscribe the concept of mental health: mental health as the absence of illness, positive mental health, and a state of equilibrium.

#### Mental health defined as the absence of illness

It was identified that this approach began in 1729 with the English medical doctor Nicholas Robinson, who stated that mental disorders indicated a change in the body that could be treated by medicine (Salaverry-García, 2012). Years later, in 1793, Scientific Psychiatry began in France due to the contributions of Philippe Pinel, which postulated that the study of the mentally ill should be based on the observation and description of the facts to provide a medical treatment according to each problem (Woods and Carlson, 1961). Subsequently, the American Psychiatric Association in 1952 published the first Diagnostic and Statistical Manual of Mental Disorders (DSM), which defines mental disorder as: “a syndrome characterized by clinically significant disturbance in an individual's cognition, emotion regulation, or behavior that reflects a dysfunction in the psychological, biological, or developmental processes underlying mental functioning” (American Psychiatric Association, 2013, p. 20.). Therefore, from this background, this paradigm establishes that a person has mental health if presents an absence of disease according to the declared symptoms.

Among the main theoretical references that recognize this paradigm are Greenspoon and Saklofske (2001), Keyes (2005), and Huppert and So (2009). Table 2 shows that 56% of the articles consider the absence of illness as part of the concept of mental health, from interdisciplinary perspectives among psychology (26 articles), philosophy (16 articles), sociology (12 articles), medicine (9 articles), psychiatry (4 articles), biology (3 articles), economics (3 articles), education (3 articles), and environmental sciences (1 article). These contributions come mainly from Europe (48%): Netherlands, Finland, Portugal, United Kingdom, Spain, Greece, Hungary, and Poland; Asia (28%): China, Hong Kong, Iran, Bangladesh, and South Korea; America (21%): Argentina, Canada, United States, Mexico, and Suriname; and Africa (3%): South Africa. Among the main mental illnesses considered in empirical studies are depression and anxiety (Kokko et al., 2013; Charalampopoulou et al., 2020; Eidman et al., 2020; Fonte et al., 2020; Romero-Pérez et al., 2020; Syrén et al., 2020; Chan et al., 2021; Wang et al., 2021). Among the main mental illnesses considered in the empirical studies are depression and anxiety (Kokko et al., 2013; Charalampopoulou et al., 2020; Eidman et al., 2020; Fonte et al., 2020; Romero-Pérez et al., 2020; Syrén et al., 2020; Chan et al., 2021; Wang et al., 2021).

**Table 2:** Results of the literature analysis according to the dominant paradigms.

**Source**	**Absence of illness**	**Positive mental health**	**State of equilibrium**
Barros et al. (2019)		X	
Baylina et al. (2018)	X	X	
Botha et al. (2019)		X	
Carlisle et al. (2009)	X	X	
Chan et al. (2018)	X	X	
Chan et al. (2021)	X		X
Charalampopoulou et al. (2020)	X		X
Chen et al. (2019)	X	X	
Chiumento et al. (2018)	X		X
de Devotto et al. (2020)		X	
de Montigny et al. (2017)		X	
de Vos et al. (2021)	X	X	
Eidman et al. (2020)	X	X	
Fonte et al. (2020)	X	X	
Galderisi et al. (2015)			X
Hendriks et al. (2017)	X	X	
Hiramoni and Ahmed (2022)	X	X	
Joshanloo (2016)		X	
Jovanović (2015)		X	
Karaś et al. (2014)	X	X	
Kavčič and Avsec (2014)		X	
Kim et al. (2020)		X	
Knoesen and Naudé (2018)	X	X	
Kocsel et al. (2022)	X	X	
Kokko et al. (2013)	X	X	
Lal et al. (2014)		X	X
Lamers et al. (2011)	X	X	
Lim (2017)	X	X	
Luijten et al. (2019)	X	X	
Lupano-Perugini et al. (2017)	X	X	
McFadden et al. (2021)	X	X	
Mehrotra and Swami (2018)		X	
Mesurado et al. (2021)		X	
Mirzakhani et al. (2020)	X	X	
Na and Lim (2020)		X	
Noorbala et al. (2012)	X	X	
Pawar (2016)		X	
Pellerin and Raufaste (2020)		X	
Petrič and Zupančič (2021)		X	
Piqueras et al. (2021)	X		
Romero-Pérez et al. (2020)	X	X	
Shin and Lim (2019)		X	X
Sikorska et al. (2021)		X	X
Singh and Junnarkar (2015)		X	
Singh et al. (2016)		X	
Sulistiowati et al. (2019)		X	
Syrén et al. (2020)	X	X	
Tedmanson and Guerin (2011)		X	
Wang et al. (2021)	X		
Webster et al. (2019)		X	
Westerhof and Keyes (2010)	X	X	
Yin et al. (2013)	X	X	

Own elaboration.

#### Mental health defined as positive mental health

This approach is based on the philosophical principles of hedonism and eudemonism. In the 4th century BC, on the one hand, the philosopher Aristippus founded the Cyrenaic school that upheld ethical hedonism based on the idea that pleasure was the supreme good (Alonso, 2017). On the other hand, Aristotle proposed the Eudemian Ethics, in which, through his book Nicomachean Ethics, he criticizes the hedonistic perspective and proposes that true happiness consists in living according to reason, in doing what is worth doing (Castelló, 1993). However, it was not until the contributions of Sigerist and Jahoda that the development of this perspective began. In 1941, Sigerist recognized that mental health is something more than the absence of mental illness (Sigerist, 1941), and in 1958, Jahoda proposed the notion of positive mental health and concepts that shaped it such as autonomy, self-realization, and mastery of the environment (Jahoda, 1959). Thus, this paradigm conceives mental health as a state of happiness or wellbeing.

In this regard, Diener adds that mental health is the subjective wellbeing expressed through a virtuous life, that is, it is the person's perception of oneself concerning virtues or normative standards (Diener, 1984). This argument together with the hedonistic and eudemonistic traditions led to the development of various perspectives on wellbeing, for example, from hedonism, wellbeing based on pleasure or the preponderance of positive emotions (Bradburn, 1969; Diener, 1984; Keyes, 2002; Diener and Tov, 2012; among others); and from eudemonism, wellbeing based on good functioning (Ryff, 1989; Keyes, 1998; Seligman, 2011; among others).

Concerning the articles reviewed, it was recognized that 86% employed positive mental health as a conceptual referent in their research (Table 2), from interdisciplinary approaches between psychology (41 articles), philosophy (28 articles), sociology (22 articles), economics (11 articles), medicine (9 articles), psychiatry (6 articles), biology (5 articles), education (5 articles); environmental sciences (1 article), and neuroscience (1 article). According to country of origin, 39% of these articles came from Europe (Netherlands, Portugal, Finland, United Kingdom, Slovenia, France, Hungary, Poland, Serbia, and Slovenia), 33% from Asia (South Korea, India, Iran, Bangladesh, China, Hong Kong, and Indonesia), 22% from America (Argentina, Canada, Brazil, United States, Mexico, and Suriname), 4% from Africa (South Africa), and 2% from Oceania (Australia).

#### Mental health defined as a state of equilibrium

This paradigm arises mainly from indications of a critique of the positive mental health perspective. Williams (2000) argued that mental health cannot be limited to happiness and wellbeing, one must consider the occasional transition to other less favorable states such as dissatisfaction, disillusionment, or depression as a normal, not pathological, process. In this sense, Seligman and Csikszentmihalyi (2000) established mental health not as a fixed state, but as an active and continuous process toward a full life; which, according to Slade (2010), is considered a recovery process in which the person makes changes that lead to a full and meaningful life, as well as a clinical outcome. In response to these contributions, Galderisi et al. (2015) proposed a new concept of mental health in line with this process; mental health is defined by these authors as a dynamic state of internal balance that allows people to use their capacities in harmony with the universal values of society.

From this historical preamble, it is recognized that this paradigm is recent and little explored in research. According to the results of the literature analysis (Table 2), it was identified that of the total number of articles analyzed, only 13% contain notions about the state of equilibrium in their theoretical frameworks when studying mental health, from various disciplines, such as psychology (5 articles), sociology (3 articles), philosophy (3 articles), psychiatry (2 articles), education (2 articles), medicine (1 article), economics (1 article), and biology (1 article). Fifty-seven percent of these articles came from Europe (Greece, Italy, Poland, and the United Kingdom), 29% from Asia (Hong Kong and South Korea), and 14% from America (Canada).

### Q3: What are the gaps identified in the conceptual notions of mental health?

Based on the sample of scientific products considered for the SLR, from different disciplines, the theoretical frameworks were analyzed to identify the conceptual references around mental health. Subsequently, these contributions were compared and categorized into paradigms. Finally, an analysis was made within each paradigm and in general, to identify areas of opportunity for the construction of an integral definition.

The analysis of the dominant paradigms from the historical approach revealed that the fragmented vision from the social and natural sciences has predominated over the years. Of the articles analyzed, only 46% integrated notions of a state of happiness or wellbeing and the absence of mental illness into the concept of mental health (Table 2). In this sense, Greenspoon and Saklofske (2001) and Keyes (2005) highlighted the importance of considering both conceptions, as they showed that they are not the same, but were related. The findings identified justify the need for a comprehensive definition that reaffirms the importance of both dominant paradigms.

From the paradigm of positive mental health, it is identified that the contributions try to adjust to the WHO's normative definition of mental health, understood as “a state of wellbeing in which an individual realizes his or her own abilities, can cope with the normal stresses of life, can work productively, and is able to contribute to his or her community” [(World Health Organization (WHO), 2001), p. 1]. Based on this definition, authors such as Keyes (2002) classified wellbeing into three aspects: (1) emotional, as the state of wellbeing; (2) psychological, which refers to working productively and fruitfully; and social, as the contribution that people make to their communities. However, there is a need to reflect on certain elements of the definition of WHO: the ability of the individual to realize his or her abilities and cope with the normal stresses of life, which seek to be addressed through the state of equilibrium paradigm.

Another gap was about how to integrate the approach of positive mental health and the absence of illness. In this sense, Keyes (2005) designed a two-continuum model to integrate both paradigms; using as a reference the definition of depression according to the DSM of the American Psychiatric Association, he identified feelings of anhedonia and functioning problems and created a parallel definition to the state of wellbeing. In this sense, in the DSM-V mental disorder is defined as a significant alteration of the individual's cognitive state, emotional regulation, or behavior (American Psychiatric Association, 2013). Therefore, from this definition, the need to distinguish between the feelings, thoughts, and behaviors of the person is located.

About the state of wellbeing, there is a tendency to define mental health as a virtuous life, that is, from virtues that serve as normative standards to judge whether a person's life is fulfilling (Diener, 1984). Thus, various virtues have been classified according to types of wellbeing (e.g., emotional, psychological, etc.). However, it has been identified that while authors such as Keyes (2002) consider life satisfaction as part of emotional wellbeing; authors such as Diener (2000) and Diener et al. (2009) argued that it is actually a cognitive process that evokes the issuance of perceptual judgments by the individual about their quality of life, so, it should not be confused with an emotional aspect; to this effect, Diener and Tov (2012) added that a person can be satisfied and not, therefore, experience high levels of positive emotions.

In that way, by including virtues to define the state of wellbeing, the influence of another conceptual notion proposed by Keyes (1998) defined as social wellbeing is located. This notion arises in the face of the criticism that wellbeing is something that goes beyond the personal level, arguing that the individual is a social being and therefore belongs to a society. In this regard, Carlisle et al. (2009) emphasized that individualistic approaches promote a vision of the self as something independent and autonomous that minimizes the social aspect of the individual and neglects interpersonal aspects. Consequently, the need arises to integrate and distinguish intrapersonal and interpersonal elements, both in notions of the state of wellbeing, the absence of disease, and the state of equilibrium.

In addition, the need to broaden the concept of the state of equilibrium is identified, which allows an understanding of the basic process through which the individual must pass in the search for personal recovery. Galderisi et al. (2015) proposed a definition that highlights the importance of the state of equilibrium, so that the individual can use his or her capabilities in harmony with social values. However, it is important to recognize what such cognitive process implies and that other authors have addressed through concepts, such as self-awareness (Wicklund, 1975), cognitive appraisal (Charalampopoulou et al., 2020), emotional intelligence (Goleman, 1995), and self-efficacy (Bandura, 1995).

Finally, it is recognized that the understanding of mental health is influenced by contributions from various disciplines and is relative to the person's cultural context. The disciplinary analysis of the scientific products revealed that the concept of mental health is approached from interdisciplinary studies between psychology, philosophy, sociology, economics, medicine, psychiatry, biology, education, environmental sciences, neuroscience, etc. However, these relationships have not been fully evidenced in the proposed definitions. About cultural influence, contributions come from various countries, mainly from Europe, Asia and America; in this sense, Galderisi et al. (2015) in their proposed definition identified that cultural differences between countries made it difficult to reach a general consensus on the concept of mental health, so, in their definition avoided statements linked to culture. These insights identify the need to broaden the concept of mental health to integrate various factors that are related to mental health and, in addition, are relative to the person and individual's cultural context.

## Discussion: Integral definition of mental health

Based on the comparative analysis of the categories designated according to the Grounded Theory approach (Strauss and Corbin, 1990), mental health was defined as the internal process of self-care, centered on the self-awareness and self-regulation of the human being, in which the person seeks to balance one's feelings, thoughts and behaviors, intrapersonal, and interpersonal, to approach an optimal state of wellbeing and absence of mental disorders based on universal values and symptoms, and in relation to biological, social, psychological, and environmental factors. Thus, mental health is a state of wellbeing that is achieved by a process of internal self-care related to external and internal factors of the person.

### Internal self-care process

This is the phase in which a person balances his feelings, thoughts, and behaviors, intrapersonal and interpersonal, through the development of his self-awareness and self-regulation skills to improve his state of mental health.

#### Self-awareness

Self-awareness is the skill of introspection and self-evaluation (Jahoda, 1959; Wicklund, 1975). Introspection is understood as the ability to focus attention on the person's internal processes, feelings, thoughts, and behaviors. Self-assessment is the ability of persons to objectively evaluate their feelings, thoughts, and behaviors, in contrast to their optimal state of wellbeing and absence of mental disorders, based on their universal values and considering their biological, psychological, social, and environmental factors, which allow them to create awareness of your state of mental health.

#### Self-regulation

**Figure 2:**
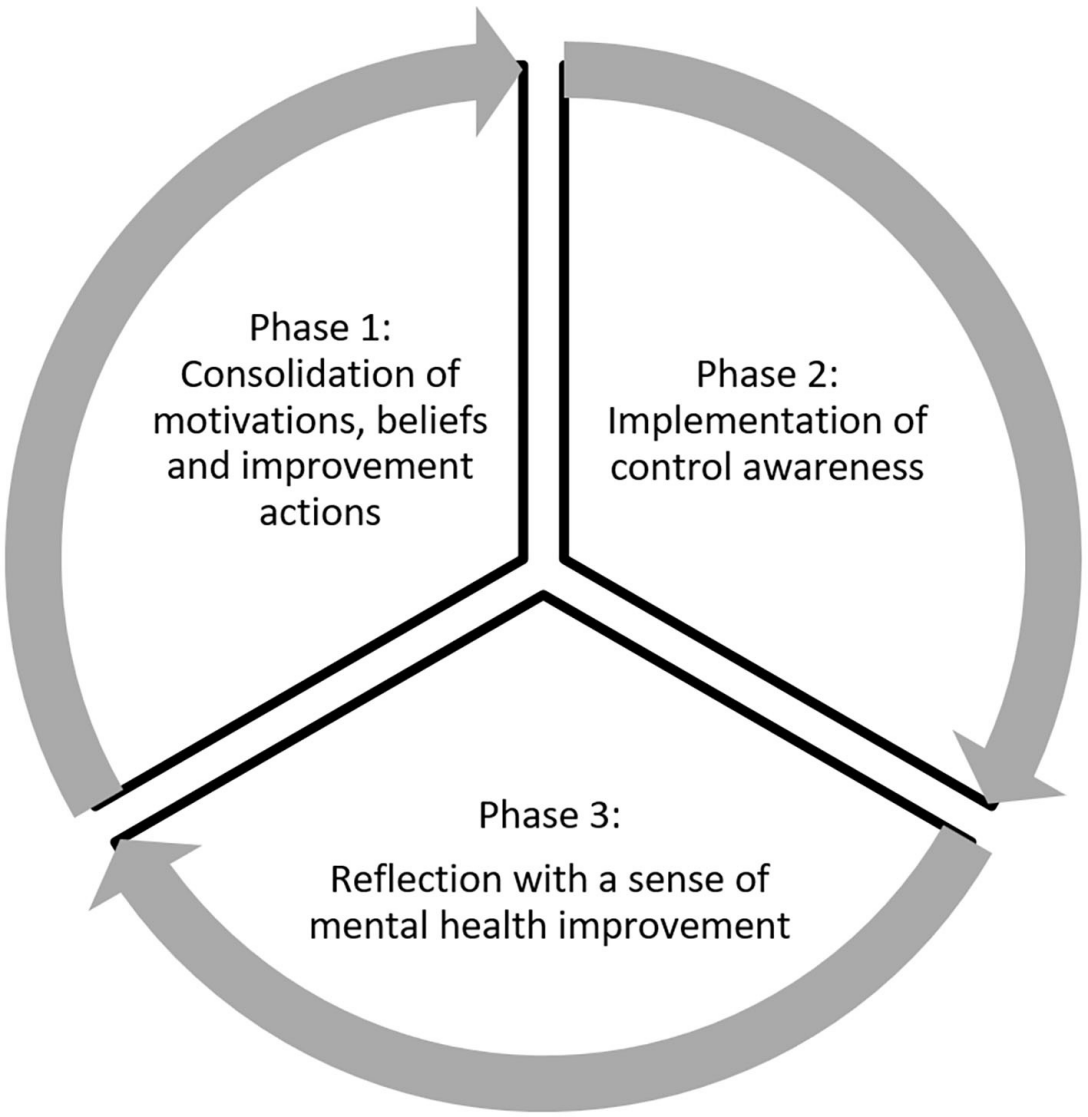
Cyclical model of self-regulation for mental health [own elaboration, adapted from Zimmerman (2000)].

Based on the contributions of Zimmerman (2000), self-regulation is redefined in the framework of mental health as the process of control and management of feelings, thoughts, and behaviors to establish and achieve individual goals toward improvement. Self-regulation is a cyclical process toward the improvement of mental health that is made up of three phases, as shown in Figure 2.

#### Consolidation of motivations, beliefs, and improvement actions (phase 1)

Once the person is aware of one's mental health status, he decides whether or not to take action to improve one's mental health depending on personal motivations and beliefs. The person may be motivated by intrinsic or extrinsic interest. Intrinsic motivation occurs when the action is performed for self-enjoyment, satisfaction inherent in the action, and internal rewards. While extrinsic motivation is given by external regulation (external rewards), introjected (ego or pride pressures), identification (utility of the activity), and integration (identification regulation completely assimilated to the self) (Ryan and Deci, 2000). In this sense, Harter (1981) and Keller and Suzuki (2004) distinguished that extrinsic values are important, but the strongest level of motivation derives from intrinsic values, and the stronger the motivation, the greater the possibility of successfully performing actions. Concerning beliefs, self-efficacy is distinguished as a key element. Self-efficacy is the confidence that the person has concerning having the necessary capabilities to obtain the desired results (Bandura, 1995); the greater the self-efficacy, the greater the possibility of setting more challenging tasks and increasing the commitment to carry them out (Locke and Latham, 1990; Bandura, 1991).

#### Implementation of control awareness (phase 2)

This occurs when attention is activated during the action to apply self-control strategies that promote success in its performance. Self-control strategies include self-instruction (describing how an activity should be performed while it is being executed), mental imagery (imagining how the activity should be performed), and identification of the essential parts of the activity for easy recall during its execution (Zimmerman, 2000).

#### Reflection with a sense of mental health improvement (phase 3)

It involves objective self-assessment of one's own performance in comparison with expected performance, and reflection to attribute causal meaning to enable rethinking actions to improve mental health.

### Optimal wellbeing state

It is the desirable mode of balance between intrapersonal and interpersonal feelings, thoughts, and behaviors based on universal personal and social values (Table 3), which allow people to enjoy a better quality of life.

**Table 3:** Universal personal and social values that designate the optimal state of wellbeing.

**Cognitive elements**	**Intrapersonal level**	**Interpersonal level**
**Feelings**	Pleasant feelings.	Pleasant feelings being with other people.
	Unpleasant feelings.	Unpleasant feelings being with other people.
**Thoughts**	Self-acceptance: unconditional approval that the individual has of oneself, recognizing one's virtues and defects.	Social acceptance: being and feeling belonging to a group.
	Self-efficacy: the individual's confidence in one's abilities to produce the desired effects.	Social actualization: confidence in the future of society, in its potential for growth and development, in its capacity to produce wellbeing.
		Social integration: assessment of the quality of relationships with society and the community.
	Life satisfaction: the individual's assessment of one's life in accordance with personal standards.	Social coherence: perception of the quality, organization, and functioning of the social world.
	Life purpose: goals, intentions, and a sense of direction that contribute to the feeling that life is meaningful.	Social identity: self-concept of the individual that comes from the knowledge of belonging to a social group.
**Behaviors**	Autonomy: self-determination, independence, and regulation of behavior according to one's own criteria.	Positive relationships: ability to build warm and trusting interpersonal relationships.
	Self-realization: performance of activities that the person considers meaningful, as they fulfill one's personal potentials.	Social contribution: belief that the person through one's actions contribute something of value to society.
	Personal engagement: state of enjoyment in which the individual experiences complete absorption in personal activities (flow state).	Social engagement: state of enjoyment in which the person experiences complete absorption in social activities (flow state).
	Personal growth: the need to actualize oneself and realize one's potentials.	Social growth: need to actualize oneself and realize one's social potential.

Own elaboration adapted from Jahoda (1959), Csikszentmihalyi and Massimini (1985), Ryff (1989), Waterman (1993), Bandura (1995), Keyes (1998, 2002), Huppert and So (2009), Diener et al. (2010), Seligman (2011), and Diener and Tov (2012).

### State of absence of mental disorders

The desirable situation of the non-existence of mental disorders, according to intrapersonal and interpersonal feelings, thoughts, and behaviors that allude to clinically recognized symptoms. Among the main mental health disorders suffered by the population worldwide are depression and anxiety; therefore, Table 4 shows the symptoms recognized by the American Psychiatric Association for these disorders.

**Table 4:** Symptoms of mental disorders (depression and anxiety).

**Cognitive elements**	**Intrapersonal level**	**Interpersonal level**
Feelings	Depression:	Depression:
	Feeling sad, down or hopeless when alone most of the time.	Feeling sad, down or hopeless most of the time when you are with others.
	Feeling of worthlessness or excessive or inappropriate guilt because you feel you have looked bad about yourself.	Feeling of worthlessness or excessive or inappropriate guilt for considering that you have looked bad to other people.
	Anxiety:	Anxiety:
	Excessive worry about various events or activities on a personal level.	Excessive worry about various social events or activities.
	Being nervous, anxious or on edge when alone.	Being nervous, anxious or on edge while being with others.
	Being irritable when alone.	Being irritable while being with others.
Thoughts	Depression:	Depression:
	Difficulty concentrating on personal activities.	Difficulty concentrating on social activities.
	Recurrent thoughts of worthlessness, suicidal or of hurting yourself.	
	Anxiety:	Anxiety:
	Uncontrolled worry caused by various events or activities on a personal level.	Uncontrolled worry brought on by various social events or activities.
	Difficulty concentrating or going blank in personal activities.	Difficulty concentrating or going blank in social activities.
Behaviors	Depression:	Depression:
	Significant decrease in interest or pleasure in all or nearly all personal activities, most of the day, nearly every day.	Significant decrease in interest or pleasure in all or nearly all social activities, most of the day, nearly every day.
	Significant impairment in important areas of personal functioning (basic needs: personal grooming, eating, etc.)	Significant impairment in important areas of social functioning (isolation).
	Anxiety:	Anxiety:
	Significant impairment in important areas of personal functioning (basic needs: personal grooming, eating, etc.)	Significant impairment in important areas of social functioning (isolation).

Own elaboration adapted from American Psychiatric Association (2013).

### Factors influencing mental health

These are aspects related to the person that has an association with one's mental health status. With respect to these aspects, biological factors (age, sex, genetics, and special conditions), psychological factors (personality traits, values, motivations, and self-regulation), social factors (educational level, gender, socioeconomic status, marital status, occupation, and family composition), and environmental factors (stressors, discrimination, bullying, challenging, hostile, among others) can be considered in accordance with the contributions of various authors [e.g., DeNeve and Cooper, 1998; Lyubomirsky et al., 2005; Keyes et al., 2010; American Psychiatric Association, 2013; Lim, 2017; World Health Organization (WHO), 2018; among others].

**Figure 3:**
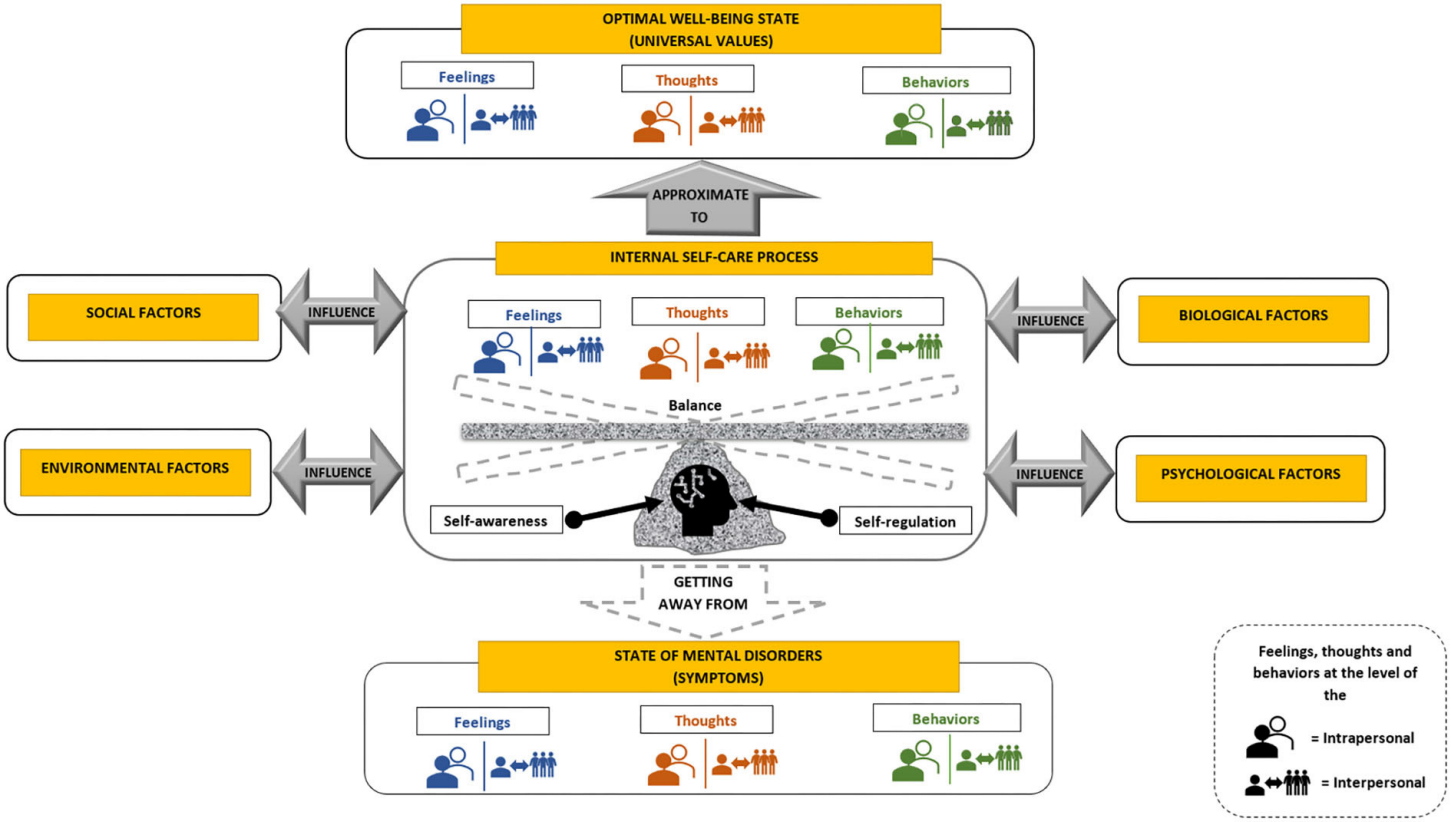
Explanatory model of the integral definition of mental health (own elaboration).

Based on this information, Figure 3 shows the explanatory model of the integral definition of mental health as a result of the Grounded Theory approach (Strauss and Corbin, 1990).

## Conclusion

The present definition and its respective explanatory model bring value to the field of mental health because it is a concept that (1) integrates contributions from various disciplines such as psychology, sociology, psychiatry, philosophy and education; (2) unites diverse paradigms such as positive mental health, mental health based on the absence of illness and mental health based on a state of balance; (3) focuses on self-care, recognized by World Health Organization (WHO) (2009) as an essential element of care for mental health; (4) seeks to empower the person to manage one's mental health problems; (5) is person-centered by considering the person's values and motivations; (6) is based on intrapersonal and interpersonal aspects, two distinct dimensions that converge to give meaning to mental health; (7) is relative to the person by taking into account various factors that vary according to development, context and personal characteristics; and (8) prevents stigmatization by focusing the concept on an internal process of personal balance, rather than a state of complete wellbeing and absence of mental disorder that could be utopian.

The present research is an approach that seeks to understand the concept of mental health from different disciplines; however, it is recognized that the keywords could exclude other disciplines that contribute to its understanding; therefore, it is recommended to analyze the concept from other disciplines not considered in the research. In addition, the complexity of the concept and its associated factors is recognized; therefore, it is suggested that future research must go deep into the study of the existing relationships between the elements that conform to the proposed explanatory model of mental health in different contexts.

## Data availability statement

The original contributions presented in the study are included in the article/Supplementary material, further inquiries can be directed to the corresponding author.

## Author contributions

MC-S conducted a systematic review of mental health paradigms. JR-M provided a context in mental health's different paradigms. The authors worked together to edit and integrate contents in the manuscript. All authors contributed to the article and approved the submitted version.

## Conflict of interest

The authors declare that the research was conducted in the absence of any commercial or financial relationships that could be construed as a potential conflict of interest.

## Publisher's note

All claims expressed in this article are solely those of the authors and do not necessarily represent those of their affiliated organizations, or those of the publisher, the editors and the reviewers. Any product that may be evaluated in this article, or claim that may be made by its manufacturer, is not guaranteed or endorsed by the publisher.

## References

[B1] Alonso P. M. (2017). Una revisión del pensamiento cirenaico: rasgos generales del hedonismo antiguo. Universidad Complutense de Madrid.

[B2] American Psychiatric Association (1952). Diagnostic and Statistical Manual of Mental Disorders. Washington, DC: American Psychiatric Association

[B3] American Psychiatric Association (2013). Diagnostic and Statistical Manual of Mental Disorders: DSM-5 (Vol. 5). Washington, DC: American Psychiatric Association. 10.1176/appi.books.9780890425596

[B4] Bakker D. Kazantzis N. Rickwood D. Rickard N. (2016). Mental health smartphone apps: review and evidence-based recommendations for future developments. JMIR Mental Health 3, e4984. 10.2196/mental.4984 26932350 PMC4795320

[B5] Bandura A. (1991). Self-regulation of motivation through anticipatory and self-reactive mechanisms in Nebraska Symposium on Motivation, ed. R. A. Dienstbier (University of Nebraska Press). 2130260

[B6] Bandura A. (1995). Self-efficacy in Changing Societies. Cambridge University Press. 10.1017/CBO9780511527692

[B7] Barros C. Fonte C. Alves S. Baylina P. (2019). Can psychosocial work factors influence psychologists' positive mental health? Occup. Med. 69, 204–210. 10.1093/occmed/kqz034 30937454

[B8] Baylina P. Barros C. Fonte C. Alves S. Rocha Á. (2018). Healthcare workers: occupational health promotion and patient safety. J. Med. Sys. 42, 1–8. 10.1007/s10916-018-1013-7 30019171

[B9] Botha B. Mostert K. Jacobs M. (2019). Exploring indicators of subjective well-being for first-year university students. J. Psychol. Africa 29, 480–490. 10.1080/14330237.2019.1665885

[B10] Bradburn N. M. (1969). The Structure of Psychological Well-being. Chicago, IL: Aldine Publishing. 10.1037/t10756-000

[B11] Busfield J. (2000). Introduction: rethinking the sociology of mental health. Sociol. Health Illness 22, 543–558. 10.1111/1467-9566.00219

[B12] Carlisle S. Henderson G. Hanlon P. W. (2009). ‘Wellbeing’: a collateral casualty of modernity? Soc. Sci. Med. 69, 1556–1560. 10.1016/j.socscimed.2009.08.029 19765875

[B13] Castelló S. F. (1993). Ética a Nicómaco. Universitat de València.

[B14] Chan R. C. Mak W. W. Chio F. H. Tong A. C. (2018). Flourishing with psychosis: a prospective examination on the interactions between clinical, functional, and personal recovery processes on well-being among individuals with schizophrenia spectrum disorders. Schizophrenia Bull. 44, 778–786. 10.1093/schbul/sbx120 28981851 PMC6007346

[B15] Chan S. L. Fonny D. H. Lau P. L. (2021). “ My struggles matter”: a phenomenological analysis of young adults recovering from major depression. J. Health Transl. Med. 24, 1–10. 10.22452/jummec.vol24no1.1

[B16] Charalampopoulou M. Bacopoulou F. Syrigos K. N. Filopoulos E. Chrousos G. P. Darviri C. (2020). The effects of Pythagorean Self-Awareness Intervention on breast cancer patients undergoing adjuvant therapy: a pilot randomized controlled trial. The Breast 49, 210–218. 10.1016/j.breast.2019.12.012 31901782 PMC7375672

[B17] Chen Y. Kubzansky L. D. VanderWeele T. J. (2019). Parental warmth and flourishing in mid-life. Soc. Sci. Med. 220, 65–72. 10.1016/j.socscimed.2018.10.026 30396119 PMC6309475

[B18] Chiumento A. Mukherjee I. Chandna J. Dutton C. Rahman A. Bristow K. (2018). A haven of green space: learning from a pilot pre-post evaluation of a school-based social and therapeutic horticulture intervention with children. BMC Public Health 18, 1–12. 10.1186/s12889-018-5661-9 29976193 PMC6034303

[B19] Csikszentmihalyi M. Massimini F. (1985). On the psychological selection of bio-cultural information. New Ideas Psychol. 3, 115–138. 10.1016/0732-118X(85)90002-911392862

[B20] Dambrun M. Ricard M. Desprès G. Drelon E. Gibelin E. Gibelin M. . (2012). Measuring happiness: from fluctuating happiness to authentic–durable happiness. Front. Psychol. 3, 16. 10.3389/fpsyg.2012.00016 22347202 PMC3273717

[B21] de Devotto R. Freitas C. P. P. Wechsler S. M. (2020). The role of job crafting on the promotion of flow and wellbeing. Revista de Administração Mackenzie. 21, 1–24. 10.1590/1678-6971/eRAMD200113

[B22] de Montigny F. Cloutier L. Meunier S. Cyr C. Coulombe S. Tremblay G. . (2017). Association between weight status and men's positive mental health: the influence of marital status. Obesity Res. Clin. Pract. 11, 389–397. 10.1016/j.orcp.2016.12.002 28007535

[B23] de Vos J. A. Radstaak M. Bohlmeijer E. T. Westerhof G. J. (2021). The psychometric network structure of mental health in eating disorder patients. Eur. Eating Disord. Rev. 29, 559–574. 10.1002/erv.2832 33949742 PMC8252750

[B24] DeNeve K. M. Cooper H. (1998). The happy personality: a meta-analysis of 137 personality traits and subjective well-being. Psychol. Bull. 124, 197. 10.1037/0033-2909.124.2.197 9747186

[B25] Diener E. (1984). Subjective well-being. Psychol. Bull. 95, 542–575. 10.1037/0033-2909.95.3.542 6399758

[B26] Diener E. (2000). Subjective well-being: the science of happiness and a proposal for a national index. Am. Psychol. 55, 34–43. 10.1037/0003-066X.55.1.34 11392863

[B27] Diener E. Lucas R. E. Scollon C. N. (2009). Beyond the hedonic treadmill: revising the adaptation theory of well-being, in The Science of Well-Being (Dordrecht: Springer), 103–118. 10.1007/978-90-481-2350-6_5

[B28] Diener E. Tov W. (2012). National accounts of well-being, in Handbook of Social Indicators and Quality of Life Research, eds. Land K. Michalos A. Sirgy M. (Dordrecht: Springer), 137–157. 10.1007/978-94-007-2421-1_7

[B29] Diener E. Wirtz D. Tov W. Kim-Prieto C. Choi D. W. Oishi S. . (2010). New well-being measures: short scales to assess flourishing and positive and negative feelings. Soc. Indic. Res. 97, 143–156. 10.1007/s11205-009-9493-y

[B30] Durkheim E. (1964). Rules of Sociological Method. Free Press.

[B31] Eidman L. Bender V. Arbizu J. Lamboglia A. T. Valle L. C. D. (2020). Bienestar emocional, psicológico y social en adultos argentinos en contexto de pandemia por covid-19. Psychologia. Avances de la Disciplina 14, 69–80. 10.21500/19002386.4851

[B32] Fink A. (1998). Conducting Literature Research Reviews: From Paper to the Internet. Thousand Oaks, CA: Sage.

[B33] Fonte C. Silva I. Vilhena E. Keyes C. L. (2020). The portuguese adaptation of the mental health continuum-short form for adult population. Commun. Mental Health J. 56, 368–375. 10.1007/s10597-019-00484-8 31583620

[B34] Galderisi S. Heinz A. Kastrup M. Beezhold J. Sartorius N. (2015). Toward a new definition of mental health. World Psychiatry 14, 231. 10.1002/wps.20231 26043341 PMC4471980

[B35] Glaser B. Strauss A. (1967). The Discovery of Grounded Theory: Strategies for Qualitative Research. Chicago, IL: Aldine Publishing Company.

[B36] Goleman D. (1995). Emotional Intelligence. London: Bloomsbury Publishing.

[B37] Greenspoon P. J. Saklofske D. H. (2001). Toward an integration of subjective well-being and psychopathology. Soc. Indic. Res. 54, 81–108. 10.1023/A:100721922788325896541

[B38] Harter S. (1981). A new self-report scale of intrinsic versus extrinsic orientation in the classroom: motivational and informational components. Dev. Psychol. 17, 300–312 10.1037/0012-1649.17.3.300

[B39] Hendriks T. De Jong J. Cramer H. (2017). The effects of yoga on positive mental health among healthy adults: a systematic review and meta-analysis. J. Alter. Complement. Med. 23, 505–517. 10.1089/acm.2016.0334 28437149

[B40] Hiramoni F. A. Ahmed O. (2022). Reliability and validity assessment of the Mental Health Continuum-Short Form for Bangladeshi Adults. Heliyon 8, e08814. 10.1016/j.heliyon.2022.e08814 35128103 PMC8803580

[B41] Huppert F. A. So T. T. C. (2009). What Percentage of People in Europe are Flourishing and What Characterises Them? Cambridge: Well-Being Institute, University of Cambridge.

[B42] Jahoda M. (1959). Current Concepts of Positive Mental Health. New York, NY: Basic Books.

[B43] Joshanloo M. (2016). A new look at the factor structure of the MHC-SF in Iran and the United States using exploratory structural equation modeling. J. Clin. Psychol. 72, 701–713. 10.1002/jclp.22287 26990960

[B44] Jovanović V. (2015). Structural validity of the Mental Health Continuum-Short Form: the bifactor model of emotional, social and psychological well-being. Person. Individ. Diff. 75, 154–159. 10.1016/j.paid.2014.11.02635584145

[B45] Karaś D. Cieciuch J. Keyes C. L. (2014). The polish adaptation of the mental health continuum-short form (MHC-SF). Person. Individ. Diff. 69, 104–109. 10.1016/j.paid.2014.05.011

[B46] Kavčič T. Avsec A. (2014). Happiness and pathways to reach it: dimension-centred versus person-centred approach. Soc. Indic. Res. 118, 141–156. 10.1007/s11205-013-0411-y

[B47] Keller J. Suzuki K. (2004). Learner motivation and E-learning design: a multinationally validated process. J. Educ. Media 29, 229–239. 10.1080/1358165042000283084

[B48] Keyes C. L. M. (1998). Social well-being. Soc. Psychol. Q. 61, 121–140. 10.2307/2787065

[B49] Keyes C. L. M. (2002). The mental health continuum: from languishing to flourishing in life. J. Health Soc. Behav. 43, 207–222. 10.2307/3090197 12096700

[B50] Keyes C. L. M. (2005). Mental illness and/or mental health? Investigating axioms of the complete state model of health. J. Consult. Clin. Psychol. 73, 539–548. 10.1037/0022-006X.73.3.539 15982151

[B51] Keyes C. L. M. Myers J. M. Kendler K. S. (2010). The structure of the genetic and environmental influences on mental well-being. Am. J. Public Health 100, 2379–2384. 10.2105/AJPH.2010.193615 20966361 PMC2978170

[B52] Kim J. Jung Y. H. Shin Y. B. Kim M. K. Eom H. Kim E. . (2020). Development and validation of a virtual reality-based training program for promoting subjective well-being. Psychiatry Investigation 17, 1207. 10.30773/pi.2020.0311 33301665 PMC8560340

[B53] Kitchenham B. Charters S. (2007). Guidelines for Performing Systematic Literature Reviews in Software Engineering. Keele University and University of Durham.

[B54] Knoesen R. Naudé L. (2018). Experiences of flourishing and languishing during the first year at university. J. Mental Health 27, 269–278. 10.1080/09638237.2017.1370635 28868951

[B55] Kocsel N. Köteles F. Galambos A. Kökönyei G. (2022). The interplay of self-critical rumination and resting heart rate variability on subjective well-being and somatic symptom distress: a prospective study. J. Psychosomatic Res. 152, 110676. 10.1016/j.jpsychores.2021.110676 34823115

[B56] Kokko K. Korkalainen A. Lyyra A. L. Feldt T. (2013). Structure and continuity of well-being in mid-adulthood: a longitudinal study. J. Happ. Stud. 14, 99–114. 10.1007/s10902-011-9318-y

[B57] Lal S. Ungar M. Malla A. Frankish J. Suto M. (2014). Meanings of well-being from the perspectives of youth recently diagnosed with psychosis. J. Mental Health 23, 25–30. 10.3109/09638237.2013.841866 24484189

[B58] Lamers S. M. Westerhof G. J. Bohlmeijer E. T. ten Klooster P. M. Keyes C. L. (2011). Evaluating the psychometric properties of the mental health continuum-short form (MHC-SF). J. Clin. Psychol. 67, 99–110. 10.1002/jclp.20741 20973032

[B59] Lim Y. J. (2017). Relationship between positive mental health and appreciation in Korean individuals. Int. J. Psychol. 52, 220–226 10.1002/ijop.12220 26390839

[B60] Locke E. A. Latham G. P. (1990). A Theory of Goal Setting and Task Performance. Prentice-Hall: Englewood Cliffs.

[B61] Luijten C. C. Kuppens S. van de Bongardt D. Nieboer A. P. (2019). Evaluating the psychometric properties of the mental health continuum-short form (MHC-SF) in Dutch adolescents. Health Qual. Life Outcomes 17, 1–10. 10.1186/s12955-019-1221-y 31640806 PMC6806567

[B62] Lupano-Perugini M. L. de la Iglesia G. Solano A. C. Keyes C. L. M. (2017). The mental health continuum–short form (MHC–SF) in the Argentinean context: confirmatory factor analysis and measurement invariance. Eur. J. Psychol. 13, 93. 10.5964/ejop.v13i1.1163 28344677 PMC5342313

[B63] Lyubomirsky S. King L. Diener E. (2005). The benefits of frequent positive affect: does happiness lead to success? Psychol. Bull. 131, 803. 10.1037/0033-2909.131.6.803 16351326

[B64] McFadden T. Fortier M. Sweet S. N. Tomasone J. R. (2021). Physical activity participation and mental health profiles in Canadian medical students: latent profile analysis using continuous latent profile indicators. Psychol. Health Med. 26, 671–683. 10.1080/13548506.2020.1757131 32319816

[B65] Mehrotra S. Swami N. (2018). Looking beyond distress: A call for spanning the continuum of mental health care. Cureus. 10, e2815. 10.7759/cureus.2815 30128219 PMC6093751

[B66] Mesurado B. Crespo R. F. Rodríguez O. Debeljuh P. Carlier S. I. (2021). The development and initial validation of a multidimensional flourishing scale. Curr. Psychol. 40, 454–463. 10.1007/s12144-018-9957-9

[B67] Mirzakhani K. Ebadi A. Faridhosseini F. Khadivzadeh T. (2020). Well-being in high-risk pregnancy: an integrative review. BMC Pregnancy and Childbirth 20, 1–14. 10.1186/s12884-020-03190-6 32912254 PMC7488451

[B68] Moher D. Liberati A. Tetzlaff J. Altman D. G. (2009). Preferred reporting items for systematic reviews and metaanalyses: the PRISMA statement. PLoS Med. 6, e1000097. 10.1371/journal.pmed.1000097 19621072 PMC2707599

[B69] Muñoz C. O. Restrepo D. Cardona D. (2016). Evolution of the concept of positive mental health: a systematic review. Pan Am. J. Public Health 39, 166–173. 27754527

[B70] Na E. Y. Lim Y. J. (2020). Influence of employment on the positive mental health of individuals with Schizophrenia living in the community. Psychiatric Q. 91, 203–208. 10.1007/s11126-019-09686-5 31811582

[B71] Noorbala A. A. Heris M. A. Alipour A. Mousavi E. Farazi G. (2012). Mental health and well-being in different levels of perceived discrimination. Iran. J. Public Health 41, 46. 23113164 PMC3481618

[B72] Pawar B. S. (2016). Workplace spirituality and employee well-being: an empirical examination. Employ. Relat. 38, 975–994. 10.1108/er-11-2015-0215

[B73] Pellerin N. Raufaste E. (2020). Psychological resources protect well-being during the COVID-19 pandemic: a longitudinal study during the French lockdown. Front. Psychol. 11, 3200. 10.3389/fpsyg.2020.590276 33424709 PMC7793808

[B74] Petrič M. Zupančič M. (2021). Personality traits predicting different aspects of subjective well-being in elderly adults. Horizons Psychol. 30, 15–25. 10.20419/2021.30.528

[B75] Piqueras J. A. Vidal-Arenas V. Falcó R. Moreno-Amador B. Marzo J. C. Holcomb J. M. . (2021). Short form of the pediatric symptom checklist-youth self-report (PSC-17-Y): Spanish validation study. J. Med. Internet Res. 23, e31127. 10.2196/31127 34855614 PMC8686478

[B76] Romero-Pérez E. M. González-Bernal J. J. Soto-Cámara R. González-Santos J. Tánori-Tapia J. M. Rodríguez-Fernández P. . (2020). Influence of a physical exercise program in the anxiety and depression in children with obesity. Int. J. Environ. Res. Public Health 17, 4655. 10.3390/ijerph17134655 32605252 PMC7369888

[B77] Ryan R. M. Deci E. L. (2000). Intrinsic and extrinsic motivations: classic definitions and new directions. Contemp. Educ. Psychol. 25, 54–67. 10.1006/ceps.1999.1020 10620381

[B78] Ryff C. D. (1989). Happiness is everything or is it? Explorations on the meaning of psychological wellbeing. J. Person. Soc. Psychol. 57, 1069–1081. 10.1037/0022-3514.57.6.1069

[B79] Salaverry-García O. E. (2012). La piedra de la locura: Inicios históricos de la salud mental. Revista Peruana de Medicina Experimental y Salud Publica. 29, 143–148. 10.1590/s1726-46342012000100022 22510921

[B80] Seligman M. E. (2011). Flourish: A Visionary New Understanding of Happiness and Well-Being. New York, NY: Free Press.

[B81] Seligman M. E. P. Csikszentmihalyi M. (2000). Positive psychology [Special issue]. Am. Psychol. 55, 5–14. 10.1037/0003-066X.55.1.5 11392865

[B82] Shalaby R. A. H. Agyapong V. I. (2020). Peer support in mental health: literature review. JMIR Mental Health 7, e15572. 10.2196/15572 32357127 PMC7312261

[B83] Shin N. Y. Lim Y. J. (2019). Contribution of self-compassion to positive mental health among Korean university students. Int. J. Psychol. 54, 800–806. 10.1002/ijop.12527 30206928

[B84] Sigerist H. E. (1941). Medicine and Human Welfare. New Haven, Yale University Press.

[B85] Sikorska I. M. Lipp N. Wróbel P. Wyra M. (2021). Adolescent mental health and activities in the period of social isolation caused by the COVID-19 pandemic. Adv. Psychiatry Neurol. 30, 79–95. 10.5114/ppn.2021.108472 PMC9881619 37082432

[B86] Singh K. Junnarkar M. (2015). Correlates and predictors of positive mental health for school going children. Person. Individ. Diff. 76, 82–87. 10.1016/j.paid.2014.11.047

[B87] Singh K. Junnarkar M. Jaswal S. (2016). Validating the flourishing scale and the scale of positive and negative experience in India. Mental Health, Religion and Culture 19, 943–954. 10.1080/13674676.2016.1229289

[B88] Slade M. (2010). Mental illness and well-being: the central importance of positive psychology and recovery approaches. BMC Health Serv. Res. 10, 1–14. 10.1186/1472-6963-10-26 20102609 PMC2835700

[B89] Strauss A. Corbin J. (1990). Grounded Theory Procedures and Techniques. Los Angeles, CA: Sage.

[B90] Suhaiban H. Grasser L. R. Javanbakht A. (2019). Mental health of refugees and torture survivors: a critical review of prevalence, predictors, and integrated care. Int. J. Environ. Res. Public Health 16, 2309. 10.3390/ijerph16132309 31261840 PMC6651013

[B91] Sulistiowati N. M. D. Keliat B. A. Wardani I. Y. Aldam S. F. S. Triana R. Florensa M. V. A. (2019). Comprehending mental health in Indonesian's adolescents through mental, emotional, and social well-being. Comprehensive Child Adolesc. Nurs. 42, 277–283. 10.1080/24694193.2019.1594460 31192730

[B92] Syrén S. M. Kokko K. Pulkkinen L. Pehkonen J. (2020). Income and mental well-being: personality traits as moderators. J. Happ. Stud. 21, 547–571. 10.1007/s10902-019-00076-z

[B93] Tedmanson D. Guerin P. (2011). Enterprising social wellbeing: social entrepreneurial and strengths based approaches to mental health and wellbeing in “remote” Indigenous community contexts. Austr. Psychiatry 19, S30–S33. 10.3109/10398562.2011.583078 21878013

[B94] Wang J. J. Lai D. W. (2022). Mental health of older migrants migrating along with adult children in China: A systematic review. Ageing Soc. 42, 786–811. 10.1017/s0144686x20001166

[B95] Wang Y. Yan J. Chen J. Wang C. Lin Y. Wu Y. . (2021). Comparison of the anxiety, depression and their relationship to quality of life among adult acute leukemia patients and their family caregivers: a cross-sectional study in China. Qual. Life Res. 30, 1891–1901. 10.1007/s11136-021-02785-6 33595826

[B96] Waterman A. S. (1993). Two conceptions of happiness: contrasts of personal expressiveness (eudaimonia) and hedonic enjoyment. J. Person. Soc. Psychol. 64, 678–691. 10.1037/0022-3514.64.4.678

[B97] Watson D. Clark L. A. Tellegen A. (1988). Development and validation of brief measures of positive and negative affect: the PANAS Scales. J. Person. Soc. Psychol. 47, 1063–1070. 10.1037/0022-3514.54.6.1063 3397865

[B98] Webster N. Oyebode J. Jenkins C. Smythe A. (2019). Using technology to support the social and emotional well-being of nurses: a scoping review protocol. J. Adv. Nurs. 75, 898–904. 10.1111/jan.13942 30585342

[B99] Westerhof G. J. Keyes C. L. (2010). Mental illness and mental health: the two continua model across the lifespan. J. Adult Dev. 17, 110–119. 10.1007/s10804-009-9082-y 20502508 PMC2866965

[B100] Wicklund R. A. (1975). Objective self-awareness, in Advances in Experimental Social Psychology (New York, NY: Academic Press), 233–275.

[B101] Williams S. J. (2000). Reason, emotion and embodiment: is ‘mental’ health a contradiction in terms? Sociol. Health Illness 22, 559–581 10.1111/1467-9566.00220

[B102] Woods E. A. Carlson E. T. (1961). The psychiatry of Philippe PINEL. Bull. Hist. Med. 35, 14–25. 13786567

[B103] World Health Organization (WHO) (2001). Basic Documents. World Health Organization.

[B104] World Health Organization (WHO) (2009). Improving Health Systems and Services For Mental Health. World Health Organization.

[B105] World Health Organization (WHO) (2018). Mental Health: Strengthening Our Response. World Health Organization. Available online at: https://www.who.int/news-room/fact-sheets/detail/mental-health-strengthening-our-response (accessed April 9, 2022).

[B106] World Health Organization (WHO) (2019). Mental Health. World Health Organization. Available online at: https://www.who.int/es/news-room/facts-in-pictures/detail/mental-health (accessed March 8, 2022).

[B107] World Health Organization (WHO) (2021). WHO Report Highlights Global Shortfall in Investment in Mental Health. World Health Organization. Available online at: https://www.who.int/news/item/08-10-2021-who-report-highlights-global-shortfall-in-investment-in-mental-health (accessed March 15, 2022).

[B108] Yin K. L. He J. M. Fu Y. F. (2013). Positive mental health: measurement, prevalence, and correlates in a Chinese cultural context, in Mental Well-being (Dordrecht: Springer), 111–132. 10.1007/978-94-007-5195-8_6

[B109] Zimmerman B. J. (2000). Attaining self-regulation: A social cognitive perspective, in Handbook of Self-Regulation, eds Boekaerts M. Pintrich P. R. Zeidner M. (New York: Academic Press), 13–40.

