# Supplementary material for: Integral definition and conceptual model of mental health: Proposal from a systematic review of different paradigms
Source: Front Sociol. 2022 Nov 25;7:978804. doi: 10.3389/fsoc.2022.978804 (PMC9732731; doi:10.3389/fsoc.2022.978804)
Supplement: Supplementary file 1 [file Table_1.DOCX]

Supplementary Material

**Supplementary Table 1.** Accepted theoretical references in the field of mental health in extended

| **Year** | **Authors** | **Contribution** | **Elements** | **Definition** |
| --- | --- | --- | --- | --- |
| 4th century B.C. | Aristippus (Alonso, 2017) | Hedonism |  | Happiness is based on pleasure as the supreme good. |
| 4th century B.C. | Aristotle (Castelló, 1993) | Eudemia |  | True happiness consists in living according to reason, "doing what is worth doing". |
| 1729 | Nicholas Robinson (Salaverry-García, 2012) | Mental illness treated by medicine |  | Any change in the mind indicated a change in the body so that affections could be treated by medicine since they implied an organic disorder. |
| 1793 | Philippe Pinel (Woods and Carlson, 1961) | Scientific Psychiatry |  | The study of the mentally ill should be based on the observation and description of the facts to give a medical treatment according to each problem. |
| 1941 | Sigerist (Sigerist, 1941) | Health beyond illness |  | Health is not only the absence of disease, but it is something positive, a joyful attitude towards life and a cheerful acceptance of the responsibilities that life places on the individual. |
| 1952 | American Psychiatric Association (American Psychiatric Association, 1952) | Diagnostic and Statistical Manual of Mental Disorders (DSM-I) |  | A mental disorder is a syndrome characterized by a clinically significant alteration in the individual's cognitive state, emotional regulation, or behavior that reflects a dysfunction in the psychological, biological, or developmental processes underlying his or her mental function. |
| 1958 | Jahoda (Jahoda, 1958) | Positive mental health | Attitudes of an individual towards oneself | Consists of accessibility to awareness (ability to introspect as needed), self-concept correction (seeing the self realistically and objectively), feeling about the self (self-acceptance, including one's shortcomings), and sense of identity (clarity of self-image). |
|  |  |  | Growth, development, and self-realization | Composed of motivational processes in which a growth motivation predominates, and investment in life through activities that it considers meaningful, beyond those done for survival. |
|  |  |  | Personality integration | Search for a unifying principle that interrelates certain areas of the psyche (ego, superego, and self). |
|  |  |  | Autonomy | Degree of independence of the individual from social influences. |
|  |  |  | Perception of reality | Perception is free of distortion, with empathy and social sensitivity. |
|  |  |  | Mastery of the environment | Adequacy in interpersonal relationships, efficiency in meeting situational requirements, ability to adapt and adjust, and efficiency in problem-solving. |
| 1969 | Bradburn (Bradburn, 1969). | Psychological well-being | Feelings | The preponderance of positive feelings over negative ones. |
| 1984 | Diener (Diener, 1984). | Subjective well-being | Life satisfaction | Determination of what is the good life according to personal standards. |
|  |  |  | Feelings | The preponderance of positive feelings over negative feelings. |
| 1988 | Watson et al. (Watson et al., 1988) | Affection | Positive affect | Enthusiastic, interested, determined, excited, inspired, alert, active, strong, proud, or thoughtful. |
|  |  |  | Negative affect | Frightened, fearful, annoyed, distressed, anxious, restless, nervous, embarrassed, guilty, hostile, or irritable. |
| 1989 | Ryff (Ryff, 1989) | Psychological well-being | Self-acceptance | Positive attitudes toward oneself. |
|  |  |  | Purpose in life | Goals, intentions, and a sense of direction that contribute to a sense that life is meaningful. |
|  |  |  | Autonomy | Self-determination, independence, and regulation of behavior according to one's own criteria. |
|  |  |  | Positive relationships | Warm and trusting interpersonal relationships. |
|  |  |  | Mastery of the environment | The individual's ability to choose or create environments suited to one' s psychic conditions; the person's ability to advance in the world and to change it creatively through physical or mental activities |
|  |  |  | Personal growth | The need to actualize oneself and realize one's potential. |
| 1993 | Waterman (Waterman, 1993) | Happiness | Experiences of personal expressiveness | Activities in which an individual experiences self-realization through the fulfillment of one´s personal potentials for the development of one´s skills and talents. |
|  |  |  | Experiences of hedonic enjoyment | It occurs when a pleasurable affect accompanies the satisfaction of needs, whether physical, intellectual, or social. |
| 1998 | Keyes (Keyes, 1998) | Social well-being | Social acceptance | Having a positive attitude towards others. |
|  |  |  | Social integration | Sense of belonging to the community. |
|  |  |  | Social contribution | It includes the belief that one is a vital member of society, with something of value to give to the world. Social contribution is similar to the concepts of effectiveness and responsibility. |
|  |  |  | Social coherence | Social coherence is the perception of the quality, organization, and functioning of the social world, and includes a concern for knowing the world. |
|  |  |  | Social actualization | It is the belief in the evolution of society and the feeling that society has potential that is being realized through its institutions and citizens. |
| 2001 | Greenspoon and Saklofske (Greenspoon and Saklofske, 2001) | Dual factor model of mental health | Disease | Mental health is the absence of mental illness. |
|  |  |  | Subjective well-being | Positive mental health based on well-being. |
| 2002 | Keyes (Keyes 2002) | Emotional well-being | Expression of well-being | Presence of positive affect and absence of negative affect. |
|  |  |  | Declared well-being | Perceived satisfaction with life. |
|  |  | Psychological well-being |  | Composed of the elements proposed by Ryff (1989): self-acceptance, purpose in life, autonomy, positive relationships, mastery of the environment, and personal growth. |
|  |  | Social well-being |  | Composed of the elements proposed by Keyes (1998): social acceptance, social integration, social contribution, social coherence and social actualization. |
| 2005 | Keyes (Keyes, 2005) | Two continuum model of mental illness and mental health | Disease | Absence of mental illness. |
|  |  |  | Flourishing | High levels of emotional, psychological and social well-being. |
| 2009 | Huppert and So (Huppert and So, 2009) | Mental health spectrum | Mental illness | Depression and anxiety. |
|  |  | Flourishing | Positive emotions | Commitment/interest and meaning/purpose. |
|  |  |  | Additional Characteristics | Self-esteem, optimism, vitality, self-determination, and positive relationships. |
| 2011 | Seligman (Seligman, 2011) | Flourishing | Positive emotion | Feel the maximum amount of pleasure and the minimum amount of pain. |
|  |  |  | Engagement | Pursuit of gratification through a state of flow in which self-consciousness is lost during an absorbing activity. |
|  |  |  | Relationships | Positive relationships. |
|  |  |  | Sense | Purpose in life. |
|  |  |  | Achievement | Related to self-realization. |
| 2012 | Diener and Tov (Diener and Tov, 2012) | Emotions and mood | Positive emotions | Joy, enjoyment, interest, euphoric, rapt, calm, relaxed, affectionate, loving, warm, happy, content, proud, grateful, optimistic, active, energetic, amazement, wonder, nostalgia, reminiscence, etc. |
|  |  |  | Negative Emotions | Anger, rage, irritation, sadness, melancholic, depressed, fearful, anxious, worried, stressed, guilt, shame, jealousy, envy, frustration, regret, rumination, pessimism, etc. |
|  |  | Cognitive judgments | Life judgments | Satisfaction, purpose, meaning, etc. |
|  |  |  | Judgments of the domains of life | Work, social relations, health, leisure, income, housing, etc. |
|  |  |  | Judgments of self-mastery | Self-efficacy, ability to help one's own group, respect from one's group, family is doing well, etc. |
|  |  | Motivational | Engagement | The person considers activities such as work, worthwhile, interesting, and involving. |
|  |  |  | Optimism | The person believes that, in general, good things will happen in the future, although recognizes that some bad events are inevitable. |
|  |  |  | Confidence | The person generally trusts others in the community. |
|  |  |  | Positive energy | The person feels energetic to work for their goals and values. |
| 2012 | Dambrun et al. (Dambrun et al., 2012) | Happiness | Self-centered psychological functioning | Perception of the self as a permanent, independent, and solid entity that favors fluctuating happiness, through positive feelings of high exaltation. |
|  |  |  | Selfless psychological functioning | Perception of the self as flexible, connected to the environment, including others, with feelings of peace of mind, low exaltation, more stable, and less dependent on external stimuli. |
| 2015 | Galderisi et al. (Galderisi et al., 2015) | Dynamic state of internal equilibrium | Basic cognitive skills | Ability to pay attention, remember and organize information, solve problems and make decisions. |
|  |  |  | Basic social skills | Ability to use one's repertoire of verbal and non-verbal skills to communicate and interact with others. |
|  |  |  | Emotion regulation | Ability to recognize, express, and modulate one's emotions. |
|  |  |  | Empathy | Ability to experience and understand what others feel without being confused with them. |
|  |  |  | Flexibility | Ability to review a course of action in the face of unforeseen difficulties or obstacles, to change one's ideas in the light of new evidence, and to adapt to changes those different times of life or contingent situations may require. |
|  |  |  | Resilience in the face of distress | Dealing with adverse life events and functioning in social roles. |
|  |  |  | Harmonious relationship between body and mind | The mind, brain, organism, and environment are strongly interconnected, and the overall experience of being in the world cannot be separated from the way the body feels in its environment. |
